# An ingestible device for automated sampling and location tracing in gastrointestinal tract

**DOI:** 10.1371/journal.pone.0327667

**Published:** 2025-07-11

**Authors:** Tao Li, Jeremy Felton, Johnathan Lewis, Qisen Cheng, Ryan Meredith, Hsueh-Tsung Lu, Alexander Benken, Partha P. Dutta, Jinhui Liao, Xiangyu D. Zhao, Aleksas Matvekas, Jason Baker, William L. Hasler, Andrew Babiskin, Ross Walenga, Lanyan (Lucy) Fang, Robert Lionberger, Manjunath P. Pai, Duxin Sun, Yogesh B. Gianchandani

**Affiliations:** 1 Center for Wireless Integrated MicroSensing and Systems (WIMS^2^), University of Michigan, Ann Arbor, Michigan, United States of America; 2 Department of Pharmaceutical Sciences, College of Pharmacy, University of Michigan, Ann Arbor, Michigan, United States of America; 3 Department of Internal Medicine, University of Michigan, Ann Arbor, Michigan, United States of America; 4 Office of Research and Standards, Office of Generic Drugs, FDA, Silver Spring, Maryland, United States of America; 5 Department of Clinical Pharmacy, College of Pharmacy, University of Michigan, Ann Arbor, Michigan, United States of America; Providence University, TAIWAN

## Abstract

Fluids sampled from the gastrointestinal (GI) tract are of interest for evaluating the bioequivalence of oral medications, and more generally for evaluating GI-related diseases, and for profiling the individual gut microbiome. Existing options for capturing multiple fluid samples from specific locations in the GI tract are limited and invasive, particularly for the small intestine. Here, we report the development of an ingestible capsule for the collection of multiple fluid samples along the GI tract; we additionally report the use of data from sensors within the capsule to determine the sampling regions. The capsule has an ingestible size of Φ14 × 42 mm^3^. Within this volume, it includes three separate cartridges that capture and retain samples within capillaries; a stepper motor for positioning the sampling cartridges at a sampling port; a 3-axis accelerometer that enables a new method of correlating sample location; a microcontroller with wireless communication and sensor data storage capabilities; and batteries to power the device. We describe *in vitro* characterization and *in vivo* tests performed with canine models that have successfully verified the capabilities of the capsule. Fluid samples from the stomach, small intestine, and colon regions of the GI tract are identified by inertial measurements taken within the capsule, and correlated to measurements of the concentration of mesalamine (a drug used for testing) and the bile salt profile in each region, respectively.

## Introduction

Sampling and analysis of fluids from multiple locations along the gastrointestinal (GI) tract, including the stomach, small intestine, and colon, are of significant interest in oral drug product development and in the investigation and diagnoses of GI conditions and diseases. For oral drug product development, it is desirable to assess drug release along the GI tract. Data obtained from *in vivo* samples help in the evaluation of drug product quality, oral drug absorption, and pharmacokinetics to ensure drug efficacy and safety [1,2]. An instrument that serves this purpose can also assist the investigation of GI conditions and diseases that rely upon the analysis of biomarkers (such as bile salts) from different regions of GI tract, which can be indicators of cancer [3,4], bacterial infections (e.g., H pylori [5,6] and C. difficile [7]), ulcer [8], GI bleeding [9], bacteria overgrowth [10], etc. Traditional sampling methods rely upon intubation using catheters [1,2] that have specially designed openings to collect fluids from multiple sites along the GI tract. Unfortunately, such catheters have only limited reach beyond the stomach into the small intestine and their use can cause discomfort and require anesthesia. As a result, catheters are not widely used for this [11,12], and a facile and reliable instrument for routine sampling of fluids from predetermined regions of GI tract remains unavailable.

An autonomous ingestible sampling device that leverages microfabricated elements and packaging technology could potentially collect and store multiple fluid samples in a manner that is noninvasive and applicable to the whole GI tract. Ingestible devices for imaging and sensing along the GI tract have a long history with both commercial options (e.g., PillCam^®^ and SmartPill^®^) and exploratory research [13–16]. The SmartPill, which carries both a motility sensor and a pH sensor, is used in clinical settings [17,18]. The PillCam, which incorporates a camera, provides images of the GI tract [19]. However, both SmartPill and PillCam are sealed devices and neither is designed to take fluid samples. In addition, neither device has the inherent means to provide regional identification of the readings that it takes. Another ingestible device, the IntelliCap^®^, has been developed for drug delivery and sensing of pH and temperature in the GI tract [20,21]. There are currently no commercially available ingestible devices for fluid sampling in the GI tract. Research prototypes that have been reported show that progress is being made toward this goal. One device was reported to perform simultaneous drug delivery and fluid sampling by thermally actuating a single piston between two reservoirs; one reservoir was used for storing the drug and the other for the collected fluid [22]. Two passive devices were reported for collection of microbiome samples from the small intestine, both using enteric coatings that block sampling inlets until dissolved at the basic pH levels in the small intestine; one device used hydrogel to absorb GI fluid samples [23], whereas the other used channels from which water was drained osmotically [24]. Another approach is to incorporate a motor within the capsule to selectively rotate a sampling port across multiple storage chambers; however, this approach has been described only conceptually in the past [12,25]. In this paper we present the first experimental results from this approach.

A localization method is also necessary to identify the GI regions where the samples are collected. A timestamp by itself is inadequate as there can be wide variations in gastric emptying time (GET) among individuals and in different body conditions [1,2]. Various localization methods for ingestible devices have been reported, using magnetic fields, radio-frequency (RF) waves, visible light, X-rays, and even magnetic resonance imaging [26–31]; all of these methods rely upon extra-corporeal equipment that is deployed, and may additionally require that the subject is immobilized. The most widely used technique is magnetic; a small permanent magnet embedded in the device is tracked by an array of magnetic sensors, such as Hall sensors, arranged outside the body near the waist. Although commercial solutions are available for locating the device in the GI tract using the sensor data, it is noteworthy that a large amount of sensor data must be processed because the device position must be identified in three-dimensions and spatially correlated to the folded segments of the GI tract [26,31]. In this work, we explore an approach that is previously unreported: we correlate the location of the sampling device with instantaneous acceleration patterns recorded by the device as it travels along the GI tract. The distinct motility patterns in stomach, small intestine, and colon, respectively [27], allow this method to identify the GI tract segments in which the samples were taken without having to use external imaging hardware or immobilize the subject.

This paper describes an ingestible device for automated fluid sampling from multiple regions along the GI tract with built-in location tracing capability, intended for applications such as those where the collected fluids can be analyzed for evaluating the effectiveness of oral drug release, for GI condition studies, and for indicators of diseases. The overall device that has been investigated, developed, and deployed in this effort is labeled PillSamp [32], and the new localization method is named the PillTrace function [33]. The device also incorporates a new method to filter and trap samples using cartridges, enhancing fluid collection while minimizing cross contamination and rejecting solid particulates. Special features in the design, fabrication, and assembly are employed to achieve fully integrated and packaged devices in a compact and ingestible form factor, and the PillSamp has been verified for sampling and tracing functions *in vivo* using canine models. The device design and implementation details are described in the Materials and Methods section. The experimental results for device implementation, benchtop testing, and *in vivo* evaluations are presented in the Results section, followed by the Discussion and Conclusions sections.

## Materials and methods

### System overview

**Fig 1:**
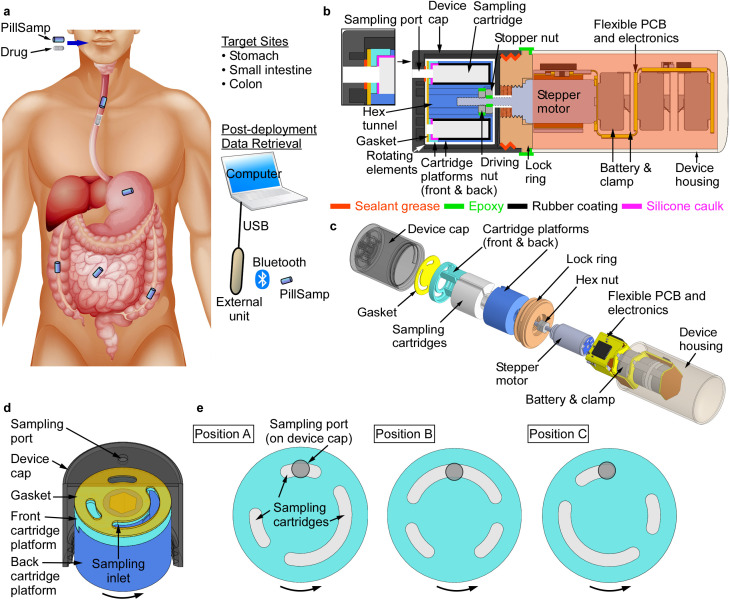
The PillSamp and its design components. **a**, Conceptual diagram of the PillSamp for fluid sampling from multiple locations along the human GI tract. Schematics of: **b**, cross-sectional view of PillSamp; **c**, exploded 3D model of PillSamp; **d**, close-up view showing integration of device cap, sampling cartridges, and front and back cartridge platforms; **e**, alignment between sampling port on device cap and sampling inlets on the front cartridge platform for sampling into each of the three cartridges.

**Fig 2:**
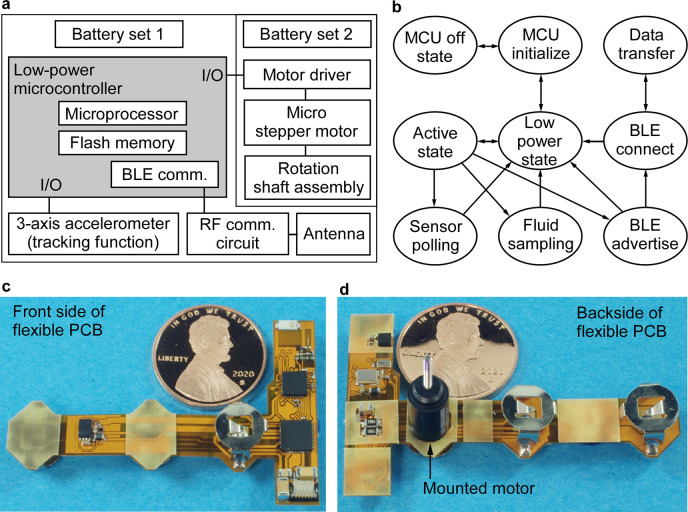
The PillSamp circuit and hardware/software functions. **a**, PillSamp circuit functional diagram. **b**, State diagram of the PillSamp software. Photos of a fabricated PillSamp flexible PCB before folding: **c**, topside; **d**, backside with a motor mounted.

As shown in Fig 1, after ingestion the PillSamp is transported along the GI tract by peristalsis. It collects fluid samples at selected sites in stomach, small intestine, and colon, and stores them on board in isolated sampling cartridges. A miniature stepper motor is used to rotate the cartridge assembly and select the cartridge to be used for sampling by aligning its corresponding sampling inlet with a single sampling port on the device cap. The electronic circuit uses a flexible printed circuit board (PCB), which is powered by three replaceable coin cell batteries and folded around the motor inside the device housing to minimize the overall device volume. A microcontroller unit (MCU) is used for device functional control, motor operation, data storage, and wireless communication (Fig 2a). The circuit also includes a 3-axis accelerometer for monitoring the device motion inside the GI tract to trace the GI segment location of the PillSamp.

The PillSamp operates autonomously once deployed. An external unit is used to initiate the PillSamp before deployment. After deployment, the same external unit retrieves collected acceleration data via a Bluetooth Low Energy (BLE) communication link, whereas the collected fluid samples are extracted from the cartridges to perform liquid chromatography-mass spectrometry (LC-MS).

### Structural design

The PillSamp includes five integral structural components (Fig 1b–1c): 1) the device housing which accommodates the electronics, batteries, and stepper motor, all assembled on a flexible PCB; 2) the front and 3) back cartridge platforms which hold three separate chambers for sampling cartridges; 4) the lock ring which facilitates device assembly and provides axial alignment; and 5) the device cap that has a single sampling port. The structural components are assembled to produce a compact and robust capsule that measures Φ14 × 42 mm^3^ in the overall size.

A number of features are used for robustness. The lock ring has an outer thread for mating with the inner thread on the device cap, and an inner thread for mating with the mounting thread of the stepper motor, allowing reliable device assembly while maintaining accurate alignment between these components that are relevant to the rotational motion. The device housing is attached to the lock ring with epoxy (Devcon^®^ 20445 5-min epoxy). The interior of the housing contains the folded flexible PCB, where it is protected from exposure to liquid. The front and back cartridge platforms lock together via key and slot features, holding the sampling cartridges in between. Fin structures inside both cartridge platforms are aligned after assembly to form three separate chambers; these chambers provide mechanical support for the sampling cartridges, ensuring proper positioning of each cartridge while also preventing cross contamination.

All structural components, including the device cap, front and back cartridge platforms, lock ring, and device housing, are made by stereolithography 3D printing (ProJet^®^ 3500) with feature resolution of 200 μm and layer resolution of 16 μm. The structural material is a biocompatible polymer, VisiJet M3 crystal, which is a translucent plastic with high durability and stability and is United States Pharmacopeia (USP) Class VI certified for medical applications [34].

### Rotational mechanism and sampling action

The PillSamp device uses a miniature stepper motor to rotate the cartridge assembly between consecutive sampling steps. The selected motor (FDM0620, Faulhaber, Schönaich, Germany) is small (Φ6 × 9.7 mm^3^), power efficient (providing maximum torque of 0.25 mNm with an 80 mA driving current), and is easily controlled using digital signals through an electronic interface chip [35]. Torque generated by the motor is transmitted to the cartridge platforms via a threaded motor shaft with two nuts: a stopper nut fixed on the motor shaft at a predetermined position, and a driving nut mated with the hexagonal hole that goes through the center of the back cartridge platforms (Fig 1b,1d). These nuts allow adjustments of the contact pressure between the device cap and the cartridge assembly. This arrangement helps to maintain a proper seal on the device port without overloading the motor. A silicone-based biocompatible sealant (Part# 24303 SG-One^TM^ Light, Anti-Seize Technology, Inc., IL, USA) is used for additional protection. The outer diameter of the cartridge assembly is minimally smaller than the inner diameter of the device cap to allow smooth rotation. The device cap isolates the rotating cartridge assembly from mechanical obstructions that might be present in the GI environment.

The front cartridge platform has three sampling inlets, one for each cartridge that is housed within a chamber formed by the front cartridge platform and back cartridge platform (Fig 1b–1d). The current implementation uses two cartridges of approximately 50 µL sample volume each, and one cartridge of approximately 100 µL sample volume. Sampling occurs when the cartridge assembly is rotated such that one of the sampling inlets aligns with the sampling port on the device cap. The design accommodates run-to-run variations in motor rotation angles. Various sampling arrangements are possible by appropriately positioning the sampling ports and adjusting the rotation motor control parameters. For example, the large sampling cartridge can be positioned to collect the fluid sample from the GI segment of the highest interest for a particular test. In another arrangement, a small cartridge can be pre-aligned with the sampling port (Position A in Fig 1e) to collect the first sample prior to motor actuation (Sample #1).

### Cartridge fabrication

The three cartridges are made from highly porous foam that provides high absorption and retention of liquid with inherent capillary force. This secure containment of the collected fluid reduces the likelihood of leakage and cross-contamination of samples. The foam also blocks the entry of particulates in the GI environment from entering the sample volume. Ester-based polyurethane (PU) open-cell foam with an average cell size of ≈200 µm (>100 ppi porosity) is selected for this work [36].

Foam sampling cartridges are made by cutting raw material of the selected foam using a hot wire thermal-cut machine (Thermocut 115/E, Proxxon Inc., NC, USA). The heated wire precisely cuts the foam without deforming the body of the foam, permitting accurate replication of the intended geometry. Each foam cartridge is coated with an impervious polymer (Multipurpose rubber coating, Plastic Dip International, MN, USA) on all surfaces except one; the uncoated surface is aligned with the corresponding sampling inlet on the front cartridge platform for sampling. The impervious coating prevents collected GI fluid from leaking out of the cartridges after sample collection. Using a highly viscous rubber solution inhibits penetration of the coating into the body of the foam before curing. Once cured, the rubber coating retains proper flexibility to allow assembly of the cartridge into the cartridge assembly.

### Circuit design

The PillSamp circuit consists of an MCU, a stepper motor and a motor driver, batteries and power regulation circuits, and a wireless communication circuit (Fig 2a). The MCU (CC2650, Texas Instruments, Dallas, TX, USA) is chosen for its small form factor (4 × 4 × 1 mm^3^), number of programmable pins available for system control, and ultra-low power consumption in both the deep sleep state and active state of operation [37]. It also contains an integrated wireless transceiver that simplifies the design of the BLE wireless communication circuit and reduces the number of discrete components required. The motor driver chip (LV8044LP, ON Semiconductor, Phoenix, AZ, USA) enables control of the stepper motor in rotation increments as small as 8° while delivering the maximum torque; it also has an ultra-low standby current of 1.0 μA to minimize power consumption when the motor is not in operation [38]. A 3-axis accelerometer (LIS2DH12, STMicroelectronics) is used for the PillTrace function to sense the XYZ acceleration of the PillSamp while it moves along the GI tract; it provides 3-axis sensing capability with ultra-low-power operation and variable data rate of 1 Hz to 5.3 kHz [39]. The motor driver and accelerometer both interact with the MCU through a standard I^2^C communication bus. For wireless communication with the external unit, a chip antenna (AH316M245001, Taiyo Yuden, Tokyo, Japan) is used with the MCU wireless transceiver for its small footprint (3.2 x 1.6 mm^2^) compared to PCB or wire antennas for BLE frequencies around 2.4 GHz.

Low-power operation is required for the PillSamp circuit to conserve battery and meet the lifetime requirement of 24–80 hr given typical deployments [40]. Multiple operating states are used to meet the low power requirement, with the PillSamp staying in a deep sleep state for majority of the deployment time and only waking up to an active state for pre-determined time intervals to perform sample collection or accelerometer reading. The deep sleep state of the PillSamp is achieved by setting the MCU into a low power mode and powering down all non-essential peripheral components between wakeup events.

Three alkaline manganese coin cell batteries (LR44, Murata Manufacturing, Nagaokakyo, Kyoto, Japan) are used to power the PillSamp. Each battery has a nominal voltage of 1.5 V and provides a 120 mAh capacity in a Φ11.4 × 5.4 mm^3^ volume. Two batteries are connected in series, along with a 320 µF shunt capacitor, to power the digital components of the PillSamp circuit. This power configuration is used to ensure a stable voltage output during high current draw events such as those during Bluetooth data transfers or accelerometer readings. The third battery is used in conjunction with a voltage booster (MCP16251, Microchip, Chandler, AZ, USA) to supply 100 mA driving current to the motor. The voltage booster not only raises the nominal voltage from 1.5 V to 3.0 V, but also permits large current to be drawn during motor rotations. In the PillSamp, this voltage booster is only activated using a control signal from the MCU during motor rotations to prevent current spikes of 20 mA magnitude and 200 Hz frequency generated by the booster from quickly draining the battery and degrading the operation lifetime.

### Software design

The PillSamp software is written in the C language for the CC2650 MCU and is designed to manage three main device functions, including motor control for fluid sampling, sensor polling to obtain acceleration data from the 3-axis accelerometer for the PillTrace function, and BLE communications for data transfer. Motor control is realized using a combination of I^2^C communication and GPIO control of the LV8044LP motor driver. The I^2^C bus is also used to communicate with the accelerometer to collect 3-axis acceleration data, which are then saved in the non-volatile flash memory of the MCU. The BLE communication is set up such that the PillSamp only advertises and attempts to connect to the external unit after the accelerometer measurements and GI fluid sampling operations have been completed. Once a BLE connection is established, the acceleration data are automatically uploaded to the external unit. All three functions are performed at pre-determined time periods, permitting the PillSamp to remain in the low power state otherwise and conserve battery life.

The operation flow for the PillSamp software is shown in Fig 2b. Immediately after the MCU is powered on, the BLE communication module is initialized, and then the PillSamp enters the low power state. After a programmed delay, the MCU enters the active state and performs one of the three main functions. In the sensor polling function, acceleration samples are collected and saved to flash memory; in the fluid sampling function, the motor driver chip is enabled, and the motor is rotated; in the BLE communication function, first the MCU sends advertising packets to attempt to connect with the external unit. If a connection is not established within 60 sec, the MCU returns to the low power state and will attempt to reconnect after a waiting period of 60 sec. When a successful connection to the external unit is established, the MCU enters the connection state and transfers the saved acceleration data. When the external unit is disconnected, the MCU returns to the low power state. The software is set up such that the MCU stays in the low power state for majority of the deployment time to extend battery life.

### LC-MS analysis

At the conclusion of each *in vivo* test, the fluids collected by the PillSamp were analyzed by LC-MS/MS. For these analyses, a Shimadzu high performance liquid chromatography (HPLC) system was coupled to an API 5500 mass spectrometer (Applied Biosystems, MDS Sciex Toronto, Canada) equipped with a turbo electrospray ionization (ESI) source. Quantification of the analytes was performed with a Xbridge C18 HPLC column (Φ4.6 mm × 150 mm, 5 µm particle size). The mobile phases were 0.1% acetic acid in purified water (A) and 0.1% acetic acid in methanol (B). A gradient separation was started with 2% mobile phase B for 1 min, then increased to 45% B at 3.5 min, and risen to 95% at 4.5 min and kept for 3 min; it was then dropped back to 2% at 8 min and kept for equilibrium for 2 min. The flow rate was set at 0.8 mL/min. The mass spectrometer was operated in a negative mode with multiple reaction monitoring (MRM) for analysis. The gas temperature was 600°C, curtain gas 45 with an ion spray voltage of −4500 V. The data were acquired and processed by Analyst software (version 6.2; Applied Biosystems, Foster City, CA, USA).

The LC-MS method used for bile acids was modified from the bile acids kit from Sigma-Aldrich. The column used was an Agilent Poroshell 120 EC-C18 column, 3 × 100 mm with 2.7 µm particles. Solvent A was 5 mM ammonium acetate plus 0.012% formic acid in water (pH 4.5), and Solvent B was 100% methanol. The gradient used was 2% to 70% B from 0 to 2 min, 70% to 95% B from 2 to 12 min, held at 95% B from 12 to 13 min, 95% B to 2% B from 13 to 14 min, then held at 2% B from 14 to 15 min. The flow rate used was 0.6 mL/min.

### Standard reference GI fluids from canine subjects

Reference GI fluids were obtained by intubation for use as comparative standards. These GI fluid samples were taken from one-year-old hounds during two separate intubation experiments. The first intubation collected stomach fluids from a fasted hound by a multi-port catheter. The second experiment used an endoscope to place the catheter in the duodenum after the pyloric sphincter for collection of bile. Stool was collected from hounds at various times during the day. No food restrictions were placed on the hounds during stool collection. Stomach and duodenal fluid samples were prepared in the same manner as samples collected by the PillSamp for drug analysis, as drug content and bile acid content were assayed from the same samples. Briefly, 10 µL of the stomach or duodenal fluid was diluted by 240 µL of acetonitrile and centrifuged to precipitate protein. The supernatant was then analyzed by LC-MS. Stool samples were diluted 1:10 mass to volume in acetonitrile and mixed by vortex before centrifugation. The supernatant was also analyzed by LC-MS.

The animal study protocol used for the collection of reference GI fluids and for *in vivo* tests of the PillSamp was approved by the Institutional Animal Care & Use Committee (IACUC) at the University of Michigan under protocol #PRO00006793.

## Results

This section presents the device implementation, benchtop testing of the PillSamp circuit, evaluation of PillSamp structural material and components, and the canine model *in vivo* evaluation of the complete PillSamp.

### Device fabrication results

The device implementation emphasized the requirements for low power consumption, small device size, and biocompatibility to meet the requirements of the target application. The steps included the fabrication of the circuit, the structural components, cartridges, and their integration.

The flexible PCB (Fig 2c–2d), with a T shape layout and a pre-folding size of 49.5 × 31.9 mm^2^, was made on the polyimide base material. Fig 2d also shows a miniature stepper motor mounted on the flexible PCB with electrical connections established between the motor and the motor driver chip on the PCB. The PCB had rigid regions with a substrate thickness of ≈0.5 mm to ensure reliable assembly of circuit components, as well as flexible regions of 0.1 mm thickness to allow it to be folded around the motor. Relative to other arrangements, this significantly reduced the overall size of the PillSamp by efficiently using the volume around the motor. The folded size of the PCB including the motor and the motor shaft was Φ12.2 × 35.4 mm^3^.

**Fig 3:**
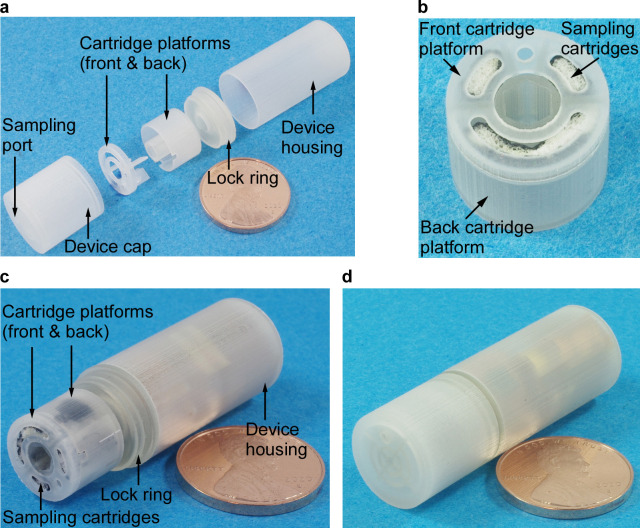
The PillSamp structural components and complete device. Photos of: **a**, 3D printed structural components for PillSamp; **b**, cartridge assembly including three foam cartridges integrated with the front and back cartridge platforms, before sealant application; assembled PillSamp, **c**, without, and **d**, with the device cap.

Fig 3a shows the 5-piece PillSamp structural components made by stereolithography 3D printing. *In vitro* cytotoxicity tests were performed to evaluate the material as described in a following section. All the structural components were produced within the designed tolerances, and with high consistency, demonstrating the rapid prototyping capability inherent to 3D printing. Furthermore, the printed components had smooth exterior surfaces, which can help passage of the PillSamp through the GI tract.

The cartridge assembly consisting of the front and back cartridge platforms and three sampling cartridges is shown in Fig 3b. Fig 3c–3d shows the fully integrated PillSamp without and with the device cap, respectively. The overall outer dimensions of the PillSamp device were Φ14 × 42 mm^3^. This was larger but still comparable to the sizes of some other commercial ingestible devices, e.g., PillCam UGI with a size of Φ11.6 × 32.8 mm^3^ [14] and was considered acceptable for swallowing by adult human and by animals such as hounds (as evidenced by our subsequent *in vivo* tests).

### PillSamp circuit tests

The fabricated PCBs were tested on the benchtop to evaluate and refine all PillSamp circuit functions, including motor control, accelerometer interrogation and data storage, BLE communication for device control and data transfer, and operation in low power state. The current consumed when performing BLE communication tasks was measured. The peak current levels during the BLE advertising phase and the subsequent BLE connection phase were 7.34 mA and 7.0 mA, respectively. These current levels were adequately buffered by the large capacitors connected in parallel to the battery power source, maintaining proper operation of the circuit while preventing the battery from exceeding its current limit. It was also verified that when BLE communication was not active, the MCU entered the low power state with the wireless radio and transceiver in the stand-by mode, resulting in <10 μA current. The motor driving capability of the flexible PCB was verified using the MCU to control the motor driver and rotate the motor. Powered by the coin cell battery and voltage booster, the motor successfully rotated at the programmed time intervals with 100 mA driving current, indicating that the coin cell battery and voltage booster could successfully support the motor operation. Benchtop operating lifetime was tested using 3 alkaline manganese coin cell batteries of 120 mAh capacity and Φ11.4 × 5.4 mm^3^ size, and was found to exceed 60 hr in a typical sampling and sensing schedule that was later used for some of the *in vivo* tests.

### Evaluation of PillSamp structural material and components

In this set of tests, the VisiJet M3 crystal polymer used for 3D printing of the PillSamp structural components was evaluated for its cytotoxicity *in vitro*; the 3D printed structural components were also evaluated *in vitro* for their viability in the GI environment.

Although the M3 polymer is USP Class VI certified for medical applications, *in vitro* cytotoxicity tests of the material were performed for verification purpose using the green fluorescent protein (GFP) expressing human fibroblasts cell line for up to 72 hr. The cytotoxicity of the material was evaluated by morphological alterations and quantitative cell viability of human fibroblast cells. Cells cultured with the material extracts did not show morphological changes or decrease of viability. The result of cytotoxicity test was negative (non-cytotoxicity), confirming the biocompatibility of the material.

The *in vitro* tests of the 3D-printed structural components were carried out by submerging a sealed assembly of all structural components without cartridges or electronics in a synthetic gastric fluid at a temperature of 37ºC for a period up to 72 hr. The synthetic gastric fluid was prepared using Dog FaSSIF/FaSSGF (Biorelevant Ltd, London, UK), a synthetic powder containing surfactants and chemicals present in the GI tract including sodium taurocholate, sodium taurodeoxycholate, lecithin, lysolecithin, and sodium oleate. A buffer solution of HCl and NaCl was made with a pH of 1.5 and NaCl concentration of 0.85 g/L and the Dog FaSSIF/FaSSGF powder was added in the buffer at a concentration of 0.16 g/L. After the *in vitro* tests, no signs of leakage or damage were observed, indicating that the components were structurally sound with no defects. This confirmed that the VisiJet M3 crystal material can withstand the GI environment, verifying its effectiveness as a structural material for the PillSamp.

### *In vivo* evaluation using hound subjects

**Fig 4:**
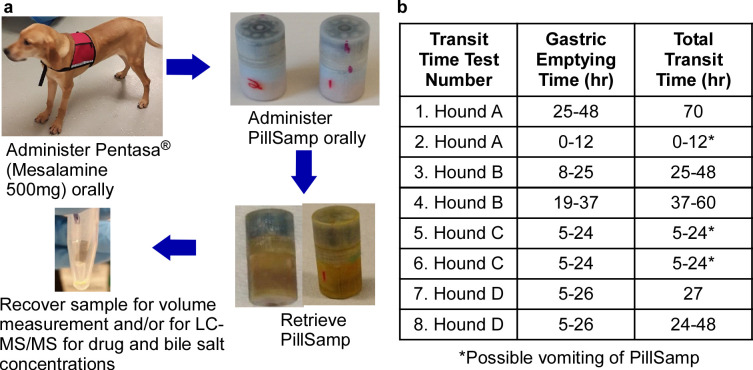
*In vivo* tests of PillSamp in hounds. **a**, *In vivo* test flow in hound. **b**, Capsule transit time in hound.

The PillSamp was extensively tested *in vivo* using mongrel hounds. These tests were performed to characterize the transit time of the PillSamp through the GI tract, evaluate effectiveness of the sealing approaches, and verify sampling and location tracing functions of the PillSamp. The general procedure performed during an *in vivo* test is shown in Fig 4a. The PillSamp was orally administered, following which, radiographs were regularly taken to monitor the approximate location of the PillSamp in the GI tract during the tests. After the PillSamp was expelled by the hound and retrieved, the device was cleaned, and the device cap was detached by unthreading from the lock ring. The cartridge assembly was removed and disassembled to allow the sample cartridges to be collected. The fluid sample collected in each cartridge was extracted by centrifuge. Volume measurement and LC-MS were then performed on the samples to analyze the concentrations of the drug and bile salt. The acceleration data were also wirelessly transferred through a BLE link to a computer for postprocessing.

#### PillSamp transit time evaluation.

Fig 4b shows a table of GET values and total transit times obtained from 8 *in vivo* experiments that were performed using 4 hounds for evaluation of PillSamp transit time. The GET was 0–12 hr in one case, 5–26 hr in five cases, and 19–48 hr in two others; the total transit time was 20–70 hr with all cases at or below 60 hrs except one. These values provided guidance for pre-programming the sampling function in the PillSamp device.

#### PillSamp *in vivo* sampling and tracing function tests.

**Fig 5:**
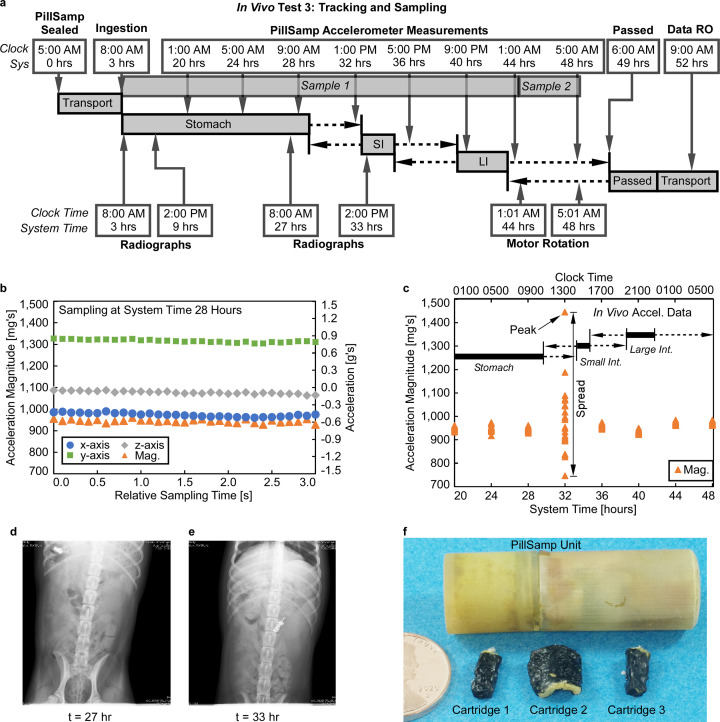
*In vivo* tests results for the PillTrace function. **a**, *In vivo* test flow in hound. **b**, recorded 3-axis acceleration data and calculated magnitude at Hour 28 during the deployment, showing modest variations that matches stomach characteristics. **c**, magnitude of recorded acceleration data through the duration of the deployment. Radiographs taken at **d**, 27 hr when the PillSamp was in stomach, and **e**, 33 hr when the PillSamp was in small intestine. **f**, Sampling foam cartridges removed from the PillSamp after the *in vivo* test. SI: small intestine; LI: large intestine. Known locations denoted with solid bar, uncertainty in location denoted with dashed line.

A series of *in vivo* tests of the PillSamp using hounds for both sampling and the tracing functions were performed. For these tests, the PillSamp was programmed to collect acceleration data and take fluid samples by rotating the internal cartridge assembly. Fig 5 shows the results from an *in vivo* test (labeled as *In Vivo* Test 3) in which a fully assembled and sealed PillSamp device was administered along with a 500 mg Pentasa^®^ (mesalamine) controlled-release capsule to a two-year old mongrel hound named Molly weighing ≈60 pounds. Pentasa has low drug release in the stomach but high drug release in the small intestine and the colon [2]. After an initial delay of 20 hr to allow time for sealing, transit, ingestion, and stomach residence, eight sets of acceleration readings were taken in 30 measurement bursts every 4 hr. The sampling port on the device cap was initially aligned to the first foam cartridge (Position A, Fig 1e), allowing a sample to be collected from the stomach. At Hour 44, the cartridge assembly was rotated to align the second foam cartridge to the sampling port (Position B, Fig 1e); it stayed in that position for 4 hr while collecting a sample from the colon, and was later moved to Position C at Hour 48. Radiographs were taken to observe the PillSamp location as it passed through the GI Tract (Fig 5d–5e). The PillSamp was expelled overnight; its overall transit time was between 43 and 49 hours. The PillSamp was interrogated, and the acceleration data were retrieved. The sampling foam cartridges were removed from the PillSamp to extract the collected samples for analysis (Fig 5f).

For the PillTrace function, recorded acceleration data (Fig 5b–5c) showed distinctive patterns in different segments of the GI tract. When the PillSamp was in the stomach, the range of variation in the acceleration magnitude was modest; however, when the PillSamp passed from the stomach to the small intestine, a distinct increase in the magnitude of the acceleration could be observed, which then diminished when the PillSamp entered the large intestine (Fig 5c). Typical results of the spread of the acceleration magnitude and peak of the acceleration from the *In Vivo* Test 3 are summarized in Table 1. Different regions of the GI tract demonstrated different acceleration magnitude patterns. It could be observed that the peak acceleration magnitude and the spread in the acceleration (30–50 milli-g) in stomach were relatively low, matching expected modest motion pattern in stomach. However, when the PillSamp moved into the small intestine, the peak acceleration magnitude and the spread (≈600 milli-g) increased significantly, which matched the characteristic high rate of transit in the small intestine and could be used as motility signatures to identify the GI segment location of the PillSamp. As the PillSamp moved into the large intestine, the peak acceleration magnitude and the spread in acceleration magnitude (20–30 milli-g), were once again diminished. These acceleration recordings were found to be typical and repeatable among all *in vivo* tests of the PillSamp, indicating a characteristic pattern of the acceleration in different segments of the GI tract that can be used to identify the GI tract segment.

**Table 1:** PillSamp location, acceleration spread and peak values of *in vivo* Test 3.

Time [hr]	Region	Accel. Spread (milli-g)	Accel. Peak (milli-g)
20	Stomach	≈30	957
24	Stomach	≈50	972
28	Stomach	≈30	960
32	Small Intestine	≈600	1442
36	Large Intestine	≈30	973
40	Large Intestine	≈20	949
44	Large Intestine	≈20	983
48	Large Intestine	≈20	983

#### Measurement of drug release and bile salts from samples collected during *in vivo* sampling tests.

As described in the last section, a series of *in vivo* tests were performed using hounds to assess the PillSamp sampling and the tracing functions. A dose of 500 mg Pentasa (mesalamine) was administered with the PillSamp device. The expelled PillSamp was retrieved to collect the samples to measure the drug concentration. The collected fluids were extracted by centrifuge and then assayed for mesalamine concentration and bile acid content.

**Fig 6:**
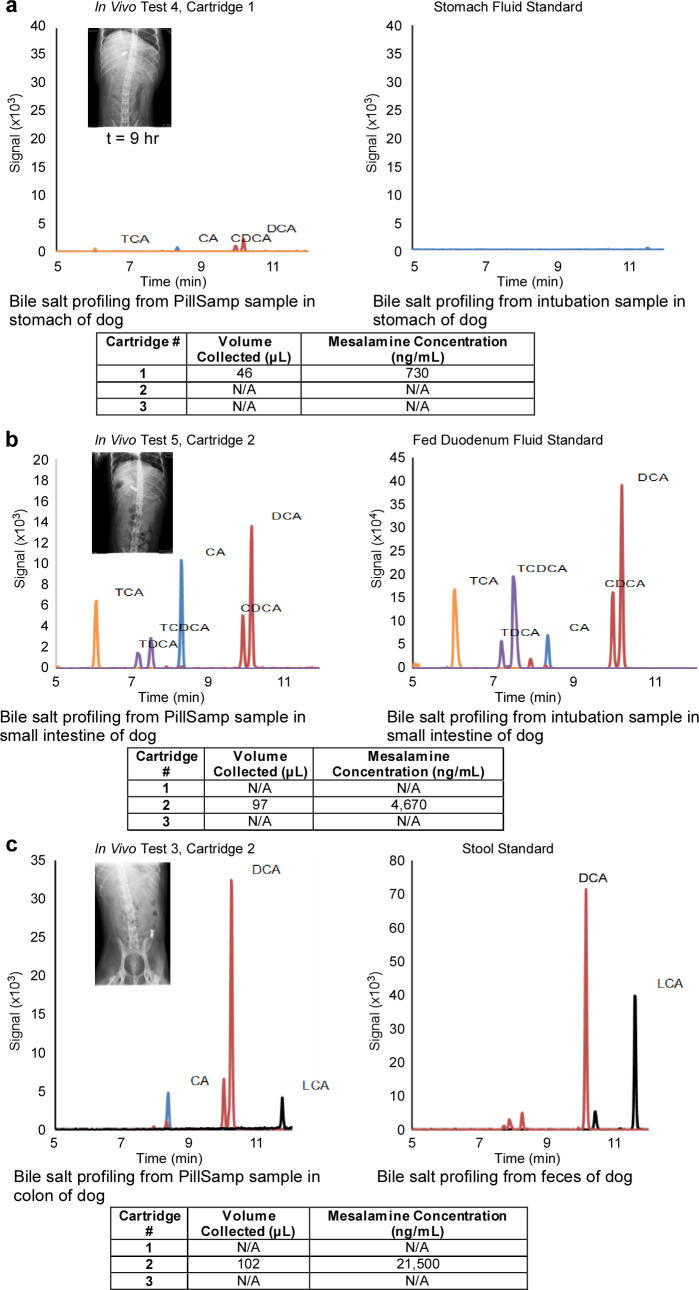
*In vivo* test results for bile salt concentration. **a**, Stomach of hound. **b**, Small intestine of hound. **c**, Colon of hound.

Fig 6a shows results from Cartridge 1 in *In Vivo* Test 4 targeting sample collection in the stomach. The concentration of mesalamine in the cartridge was 730 ng/mL. This sample had a low bile salt profile, which, in combination with the radiographs, indicates that the sample was from the stomach.

Fig 6b shows results from Cartridge 2 in *In Vivo* Test 5 targeting sample collection in small intestine. The hound used in this experiment was the same as in *In Vivo* Test 3, and there was much higher mesalamine concentration in both experiments at levels expected for *in vivo* dissolution. The bile acid profile of this sample matches the fed state fluid from the duodenum. This indicates that the sample came from the duodenum. The radiographs also indicate that the sample was taken when the PillSamp was in the duodenum.

Fig 6c shows results from Cartridge 2 in *In Vivo* Test 3 targeting sample collection in large intestine. The mesalamine concentration matches the expected level in dogs and therefore the value is plausible. The bile acid profiles also help confirm the origin of the samples. The presence of lithocholic acid (LCA) in the sample indicates it is likely from the colon, which is also confirmed by the radiographs.

## Discussion

The PillSamp device reported in this work is compared in Table 2 to other devices reported in research literature that also provided *in vivo* results for GI sampling. Importantly, PillSamp was able to collect up to three GI tract fluid samples stored in individual foam cartridges housed inside the PillSamp device while other devices were intended for one sample only. Selection between foam cartridges in PillSamp was achieved by incorporating a stepper motor to rotate the capsules in order to align them with the sampling port of the device cap. Sample collection was accomplished by capillary action. The overall size of the PillSamp device was similar to other devices described in Table 2 with a diameter of 14 mm and length of 42 mm. The PillSamp was capable of wireless communication based on the standard BLE protocol with broad compatibility instead of the specially developed RF communication method used in one of the other devices.

**Table 2:** Comparison of ingestible GI tract sampling devices with *in vivo* results.

Device	Sampling Action	Fluid Sampling Capability	Commu-nication	Location Tracing	Capsule Size	Animal Species
PillSamp (this work)	Capillary action	1 × 130 µL + 2 × 50 μL (triggered by stepper motor)	RF (BLE)	Embedded micro-accelerometer	Ø14 × 42 mm^2^	Canine/ Hound
Cui capsule [22]	Vacuum sorption	1 × 262 μL (triggered by MEMS calorific element)	RF	Embedded magnet	Ø11 × 30 mm^2^	Porcine
Nejad pill [24]	Osmotic pumping	1 × 120 µL (triggered by dissolution of enteric capsule)	No	Embedded magnet	Ø9 × 24 mm^2^	Porcine & Macaque

The acceleration data successfully collected from the GI tract deployment represent the first effort in exploring the use of the acceleration pattern for location tracing inside the GI tract. The results suggest that it is feasible to identify motion patterns in different segments of the GI tract using an accelerometer and, therefore, identify the segment location of the PillSamp during its GI tract transit. The acceleration data demonstrate the motility within different regions of the GI tract and can be used to allow the PillSamp to autonomously identify which region it is in, permitting sampling in different regions of the GI tract without requiring external control or radiograph tracing. It is also notable that the recorded acceleration of the PillSamp is not affected by the activity of the hound, diminishing the need for an external reference unit that is worn by the hound to monitor its body acceleration. This is because the GI tract presents a highly damped environment that protects the PillSamp from external shock, and the further encapsulation of the accelerometer within the PillSamp provides additional isolation.

A significant finding in these experiments was that the spread (≈600 milli-g) and the peak of the acceleration magnitude of the PillSamp increased significantly in the small intestine. This may suggest a unique characteristically high rate of transit in the small intestine. To confirm if this indeed reflects the transit pattern in the small intestine, we also used PillCam to visualize the Pentasa movement in the small intestine vs. stomach. As shown in the supplemental materials, the Pentasa capsule was disintegrated into granules in the stomach (S1a Fig in S1 File), passed through pylorus (S1b, S1c,S1d Fig in S1 File), and entered small intestine (S1e Fig in S1 File). Indeed, the Pentasa granules transited into the small intestine in a much faster back-and-forth motion than that in the stomach. The increased spread and peak of the acceleration magnitude recorded by PillSamp may have reflected these movements.

## Conclusions

In this paper, a new ingestible PillSamp device was designed and tested in a canine model. The reported device is capable of collecting three fluid samples to determine concentration of drug and bile salts in the three regions of the GI tract (stomach, small intestine, and colon). Compared to the existing alternative approach using GI intubation, the PillSamp device is less invasive, does not require constant medical supervision, and causes less subject discomfort. Future work will focus on optimization of the engineering design of the device for reducing the overall size of the device and the fabrication and assembly processes for improved manufacturability. Further extensive testing of the optimized device will help evaluate and enhance the sampling and location tracing functions of the device in diversified usage scenarios, leading toward clinical trials of the device in the contexts of drug product development as well as the understanding and diagnosis of GI conditions and disease.

## Supporting information

S1 File Supporting information on: *In vivo* visualization of the transit pattern using PillCam; Device assembly; PillTrace results from additional *in vivo* tests. (PDF)

S2 File Data file of PillTrace results from *in vivo* tests. (XLSX)
