# Supplementary material for: An ingestible device for automated sampling and location tracing in gastrointestinal tract
Source: PLoS One. 2025 Jul 11;20(7):e0327667. doi: 10.1371/journal.pone.0327667 (PMC12250150; doi:10.1371/journal.pone.0327667)
Supplement: S1 File — (PDF) [file pone.0327667.s001.pdf]

Supporting information

In vivo visualization of the transit pattern using PillCam

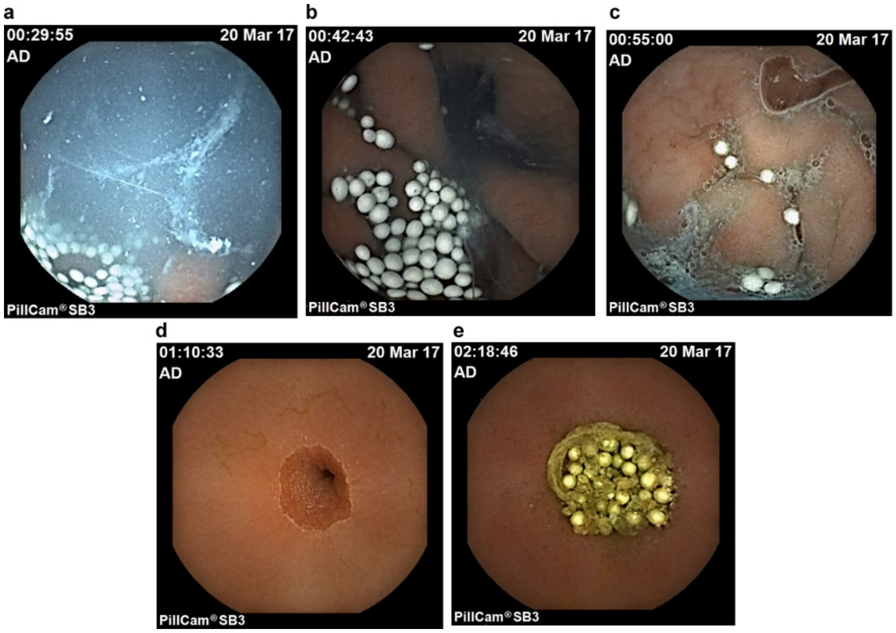

Fig. S1: *In vivo* visualization of Pentasa (mesalamine 500mg) disintegration/dissolution and transit using PillCam video capsule endoscopy in fasted dog. a, Pentasa capsule disintegrated into granules in the stomach. **b**, Pentasa granules about to enter pylorus. **c**, Most Pentasa granules left stomach. **d**, All Pentasa granules left stomach. **e**, Pentasa granules transit in back-and-forth motion in the small intestine.

The transit, disintegration, and dissolution of mesalamine (500 mg, Pentasa) were visualized using a PillCam video capsule endoscopy to verify the existence of a unique and characteristically high rate of transit in the small intestine. The elevated transit rate was implied by the PillSamp measurements as well, as indicated by the significantly increased peak and spread of the acceleration magnitude recorded in the small intestine. As shown in Fig. S1, the Pentasa capsule disintegrated into granules in the stomach (Fig. S1a), passed through pylorus (Fig. S1b,c,d), and entered small intestine (Fig. S1e). In the small intestine, the Pentasa granules exhibited much faster back-and-forth motion than that in the stomach. The increased spread and peak of the acceleration magnitude recorded by PillSamp were consistent with these PillCam observations.

Device assembly

The assembly process for the PillSamp starts with construction of the cartridge assembly, including the assembly of the front and back cartridge platforms with the sampling cartridges. A fine layer of silicone caulk (Almond Silicone II® 28055, General Electrics, MA, USA) is applied along the perimeter of the inner surface of the front cartridge platform around the sampling inlets. This is followed by insertion of the rubber-coated foam cartridges, which form a removable bond with the silicone caulk. After all cartridges are inserted into the front cartridge platform, a biocompatible silicone-based sealant grease (Part# 24303 SG-One™ Light, Anti-Seize Technology, Inc., IL, USA) is applied to fill any gap and open space in the assembly. The back cartridge platform is then inserted with the key and slot features fitting together; a low-viscosity adhesive (Loctite® Superglue, Henkel, Düsseldorf, Germany) is applied along the contacting edges to bond the front and back cartridge platforms (Fig. 3b). The low viscosity of the adhesive ensures that the gaps between the cartridge platforms are entirely sealed. The driving nut is then inserted into the hexagonal hole of the back cartridge platform and held in place with a high-viscosity epoxy (20445, Devcon ltd., CA, USA). Next, a gasket is mounted to the front cartridge platform using the low-viscosity adhesive (Fig. 1b). The gasket is cut from 5 mil-thick sheets of polyimide (500HN, DuPont, DE, USA), which is chemically inert and mechanically robust. Sealant grease is applied between the device cap and the gasket on the front cartridge platform around the sampling port.

For attaching the motor to the flexible PCB, the stopper nut is threaded onto the motor shaft and fixed at a predetermined position, allowing the stopper nut to be seated within the recess in the back cartridge platform after finishing assembly. Next, the motor is mounted to the backside of the flexible PCB using conductive epoxy (Duralco™ 120, Cotronics Corp., NY, USA).

Additional high-viscosity adhesive is applied at the motor base to secure the stepper motor in place. The cartridge assembly is then threaded onto the motor shaft until the driving nut makes contact with the stopper nut. The flexible PCB is then folded around the motor to form a compact geometry so that it can be inserted into the device housing (Fig. 1b-c). The open space in the device housing surrounding the folded flexible PCB is filled with silicone-based sealant grease as a precaution against leakage into the device housing. The device cap is threaded onto the lock ring with the sealant grease applied on the thread for sealing. Finally, the lock ring and the device housing are bonded and sealed with the high-viscosity adhesive, resulting in a fully assembled PillSamp (Fig. 3c-d). The lock ring reliably aligns and connects the fluid end and the electronics end of the device. The overall structure effectively maintains isolation between the fluid end and electronics end to prevent damage of the PillSamp circuit from leakage.

PillTrace results from additional *in vivo* tests

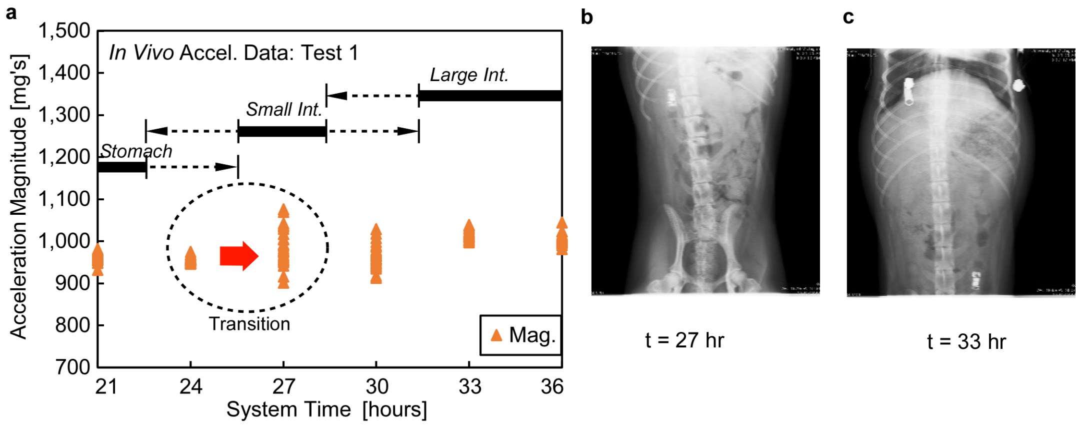

Fig. S2: Results from *in vivo* Test 1 for the PillTrace function. a, Magnitude of recorded acceleration data during the deployment. Radiographs taken at **b**, 27 hr when the PillSamp was in small intestine, and **c**, 33 hr when the PillSamp was in large intestine. Known locations denoted with solid bar, uncertainty in location denoted with dashed line.

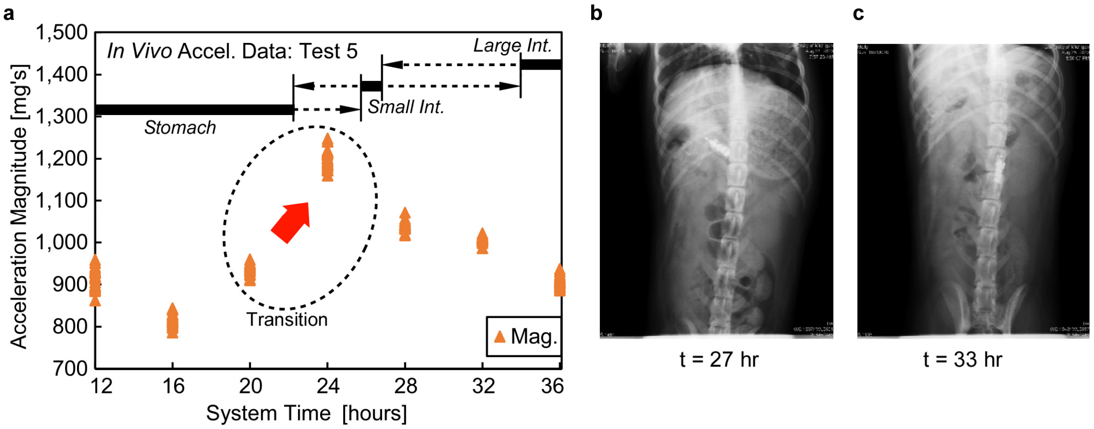

Fig. S3: Results from *in vivo* Test 5 for the PillTrace function. a, Magnitude of recorded acceleration data during the deployment. Radiographs taken at **b**, 27 hr when the PillSamp was in small intestine, and **c**, 33 hr. Known locations denoted with solid bar, uncertainty in location denoted with dashed line.

Besides Fig. 5 that shows the results for the PillTrace function from *In Vivo* Test 3, Fig. S2 and S3 show the results from additional *in vivo* tests for the PillTrace function performed using the same procedure. Figure S2 shows the results from *In Vivo* Test 1, which was performed on a two-year old mongrel hound named Josie weighing ≈60 pounds. Figure S3 shows the results from *In*

Vivo Test 5, which was performed on a two-year old mongrel hound named Molly weighing ≈60 pounds. Radiographs were taken in each test to observe the locations of the PillSamp in both tests as shown in Fig. S2 and Fig. S3, respectively. Both tests showed a transition that was similar to *In Vivo* Test 3 in the acceleration signals that were recorded as the PillSamp moved from the stomach to the small intestine: the peak acceleration magnitude increased substantially.
